# Anemia and Associated Factors among Public Elementary School Children in Asella Town, Southeast Ethiopia: A Facility-Based Cross-Sectional Study

**DOI:** 10.1155/2024/1519382

**Published:** 2024-04-24

**Authors:** Ararso Hordofa Guye, Kasim Hansa, Kasahun Ketema, Meseret Moroda, Dame Banti Shambi

**Affiliations:** Department of Public Health, College of Medicine and Health Sciences, Salale University, Fiche, Ethiopia

## Abstract

**Background:**

Anemia has a negative impact on school children, including poor physical growth and reduced mental performance. Children show poor attentiveness, behavior, and memory and reduced school performance. There is limited evidence of the magnitude of anemia and associated factors in school-age children in Ethiopia, including the study area.

**Objective:**

To assess the magnitude of anemia and associated factors among public elementary school children in Asella Town, Southeast Ethiopia, in 2022.

**Methods:**

A school-based cross-sectional study was conducted in Asella Town from April 5 to May 5, 2022. A total of 442 school children aged 7–14 years were included in the study using the multistage sampling method. Data were collected using a pretested and semistructured questionnaire through a face-to-face interview technique. The hemoglobin concentration was determined by using the HemoCue 301+ analyzer. Anthropometric data and stool examinations were collected from participants. Data were entered into EpiData version 4.6, transported, and analyzed by Statistical Package for Social Sciences version 26. Bivariable and multivariable logistic regression analyses were carried out. Adjusted odds ratios along with their 95% confidence interval were used, and a *p* value of ≤0.05 was used for declaring statistical significance.

**Results:**

A total of 435 students with a mean age and standard deviation of 10.77 ± 2.21 years participated in the study. The magnitude of anemia was 78 (17.9%), with a 95% CI (14.3, 21.47). Of the participants, 63 (14.5%) were mild anemic and 15 (3.4%) were moderately anemic. Children whose mothers have no formal education (AOR = 3.94, 95% CI: 1.89, 8.21), underweight children (AOR = 3.83, 95% CI: 1.98, 7.40), and parasites in their stool (AOR = 3.72, 95% CI: 1.50, 9.20) were significantly associated with anemia in school-age children.

**Conclusion:**

Anemia among school-age children was found to be a mild public health problem. Uneducated mothers, intestinal parasite infections, and underweight children were found to be determinants of anemia among school-age children. Health professionals should provide health education for mothers about child-feeding practices and the consumption of dietary sources of iron.

## 1. Introduction

Anemia is a condition in which the number and size of red blood cells, or the hemoglobin concentration, fall below an established cut-off point, consequently impairing the capacity of the blood to transport oxygen around the body [1, 2]. Anemia is an indicator of both poor nutrition and poor health [1]. When hemoglobin (Hob) levels fall below the population-specific Hob threshold, it is considered a public health issue [3] and can be classified as no, mild, moderate, and severe public health problems when the prevalence is ≤4.9%, 5.0–19.9%, 20.0–39.9%, and ≥40%, respectively [2, 4].

The World Health Organization (WHO) definitions of anemia vary by age, and the sixth WHO defines anemia in children aged 5–11 years as hemoglobin <11.5 g/dl and in children aged 12–14 years as hemoglobin <12 g/dl [5]. Among school-age children, it could be due to inadequate consumption of nutrient-rich foods, dietary taboos, lack of access to health care, and inefficient utilization of available micronutrients due to cause of infections and parasitic infestations, among other reasons [4, 6, 7]. Anemia is a worldwide public health problem affecting both developing and developed countries, with major negative impacts on human health and social and economic development [8].

Globally, around 1.62 billion people are affected by anemia, which accounts for more than 24.8% of the world population, and from 30 to 50% of anemia is caused by iron deficiency. Iron deficiency anemia (IDA) resulted in 273,000 deaths in the world, and 97% of deaths occurred in developing countries. According to a WHO report, the global anemia prevalence of school-age children (SAC) was 25.4%, followed by preschool children under 5 years old (47.4%) [9]. Anemia results from one or more of the following processes: defective red cell production, increased red cell destruction, or blood loss [9]. Iron is necessary for the synthesis of hemoglobin [9]. Iron deficiency is thought to be the most common cause of anemia globally, but other nutritional deficiencies such as folate, vitamin B12, and vitamin A, acute and chronic inflammation, parasitic infections, and inherited or acquired disorders that affect Hob synthesis, red blood cell production, or red blood cell survival can all cause anemia [10, 11]. At least half the burden of anemia is associated with iron deficiency [11].

Africa and Southeast Asia are the regions that present the highest prevalence of this disease [12]. A study conducted in eight countries in Africa and Asia showed that 12–58% of SAC are suffering from anemia and 12%, 41%, 54%, 57%, and 58% in Malawi and Kenya, Ghana, Mozambique, Tanzania, and Mali, respectively, and 30% in Vietnam and Indonesia [13–15]. More than 40% of SAC in developing countries suffer from anemia, and it is considered a severe public health problem. Sub-Saharan African countries shared a greater burden of the problem [16].

Children are particularly vulnerable to iron deficiency anemia (IDA) because of their increased iron requirements during periods of rapid growth, especially in the first 5 years of life [17]. Public health interventions to ameliorate micronutrient malnutrition in preschool and school-age children include the promotion of dietary diversification to include foods rich in highly absorbable vitamins and minerals, anthelminthic treatment, mass fortification of staple foods and condiments, home (point-of-use) fortification of foods, and provision of micronutrient supplements [18].

Iron deficiency, a common form of nutritional deficiency during childhood, results from sustained negative iron balance, which is caused by inadequate dietary intake, absorption or utilization of iron, increased iron requirements during the growth period, or blood loss due to parasitic infections such as malaria, soil-transmitted helminth infestations, and schistosomiasis. In later stages of iron depletion, the hemoglobin concentration decreases, resulting in anemia [18]. The main risk factors for IDA include a low dietary intake of iron or poor absorption of iron from diets rich in phytates or phenolic compounds [4]. Anemia has a negative impact on school children (SC), including poor physical growth, and children show poor attentiveness, behavior, and memory and reduced school performance [19]. Children who suffer from anemia have reduced mental (cognitive) performance, poor attention span and learning ability in children, delayed psychomotor development, impaired work performance, and increased morbidity because of reduced resistance (low tolerance) to infection; in addition, they experience impaired coordination of language and motor skills, equivalent to a 5–10 point deficit in intelligence quotient [9, 12, 20–22]. Anemia has detrimental effects on the cognitive performance and physical development of school children, which can lead to low productivity, limited work capacity, delayed physical growth, and poor academic achievement [17, 23, 24].

Studies conducted in Ethiopia documented that factors that affect anemia in school-age children were mother occupation status, father occupation status, maternal education, father education, family size, parasites in the stool, stunted, and underweight [12, 15, 16, 24–29]. The Ethiopian government has been working to address the nutritional concerns of SAC, mainly by integrating nutritionally sensitive interventions such as conducting regular monitoring of the nutritional status of school-age children/students together with biannual deworming and water hygiene and sanitation (WASH) programs in schools [30], mass fortification of staple foods and condiments, home (point-of-use) fortification of foods, initiating a homegrown school feeding program for school-aged children, and provision of micronutrient supplements and weekly iron and folic acid supplementation (WIFAS) [19, 31].

There is limited evidence on the magnitude of anemia and associated factors in school-age children in Ethiopia, including the study area. Therefore, this study aimed to assess the magnitude of anemia and identify associated factors among SC attending public elementary schools in Asella Town, Southeast Ethiopia.

## 2. Methods and Materials

### 2.1. Study Design, Period, and Setting

A school-based cross-sectional study design was conducted in public elementary schools in Asella Town from April 5 to May 5, 2022. Asella is the capital city of the Arsi Zone, which is located at a distance of 165 km from Addis Ababa, Southeast Ethiopia. The estimated population of Asella Town in 2022 is 113,445, of whom 57,290 are male and 56,155 are female. According to the education office of the town, Asella Town has a total of 27 primary schools (9 governmental and 18 private) during the study. In the academic year 2022, there were a total of 11654 students from grades 1–8. From this, about 46.6% of students were female. Regarding the health facilities of the town, there is one referral hospital, two governmental health centers, eight health posts, fourteen medium clinics, and thirty pharmacies and drug stores.

#### 2.1.1. Source Population

The source population for this study was all SC (aged 7–14 years) in public elementary schools in Asella Town.

#### 2.1.2. Study Population

The study population was all school children (aged 7–14 years) in the randomly selected elementary schools in Asella Town.

#### 2.1.3. Study Unit

Individual students aged 7–14 years were selected from the roster in randomly selected schools.

### 2.2. Eligibility Criteria

#### 2.2.1. Inclusion Criteria

All children in the age group of 7 to 14 years in selected elementary schools who had been living in the study area for more than six months were included.

#### 2.2.2. Exclusion Criteria

Students treated for intestinal schistosomiasis and/or soil-transmitted helminths in the past month and children with deformity were excluded.

### 2.3. Sample Size Determination

The sample size for the first objective was determined using Epi Info 7 by consideration of the following assumptions: prevalence of 15.5% [12], 95% confidence level, 5% margin of error (d), design effect of 2, and nonresponse rate of 10%.

(1) $$\begin{array}{c} n=\frac{{Z\alpha /2}^{2}P\left(1-P\right)}{d^{2}}\\ =\frac{{\left(1.96\right)}^{2}0.155\left(1-0.155\right)}{0.05^{2}}\\ n=201. \end{array}$$

By comparing the first and second objectives, the maximum sample size was 201 from the first objective, and by considering the design effect of 2 and 10% of the nonresponse rate, the final sample size was 442. The sample size was calculated for the second objective (selected associated factors) using Epi Info, assuming a two-sided confidence level = 95%, power = 80%, and ratio = 1 (Table 1).

### 2.4. Sampling Procedure

**Figure 1:**
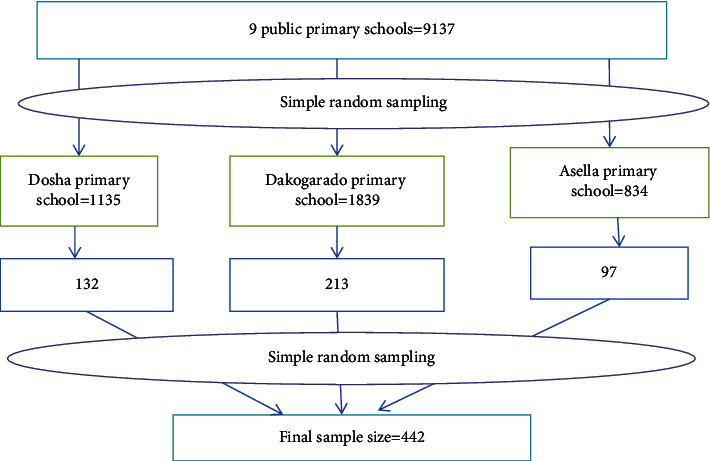
Schematic presentation of the sampling procedure for the assessment of anemia and associated factors among public elementary school children in Asella Town, 2022.

A multistage sampling procedure was used to choose the study participants. In the beginning, governmental elementary schools in the town were registered. Then, three governmental schools were randomly selected from 9 elementary schools found in Asella Town using the lottery method. Then, the study participants in the target age group enrolled in the selected schools were identified and the total number of students was obtained from each school director's office. Based on the number of students in each school and class/grade, the sample size was proportionally distributed across the selected schools. Next, the number of participants required to be enrolled was allocated proportionally based on the number of students in each class and grade level. Finally, the allocated sample size was selected from each school by a simple random sampling procedure using computer-generated random numbers (Figure 1).

### 2.5. Data Collection Tools and Procedures

Questionnaires were adapted and modified from studies [12, 16, 26]. The questionnaire contains variables on sociodemographic characteristics, educational status, and factors associated with anemia. Three BSc nurses and two medical laboratory technologists were recruited for data collection, and one senior laboratory technologist checked for discrepancies between the two data collectors. One health officer was recruited as supervisor, and the investigators were supervised for leading the whole data collection process. Data were collected through a face-to-face interview in the language of the respondent by using a semistructured Afaan Oromo version questionnaire and laboratory investigation.

#### 2.5.1. Anthropometric Measurements

Anthropometric data were collected by recording the age, weight, and height of the participants according to the WHO guidelines [33]. A portable digital weight scale with a capacity of 120 kg was used, and each child was weighed with slight clothing and barefoot and recorded to the nearest 0.1 kg. Height was measured in the Frankfurt position (head, shoulder, buttocks, knee, and heels touching the vertical board) by using a portable wooden height board (audiometers) with a sliding head bar, and then, height was measured to the nearest 0.1 cm. Every measurement was calibrated by placing standard calibration weights on the scale to ascertain accuracy. Measurements of weight and height were taken twice, and the mean was recorded [33].

To determine the nutritional status of school children, the anthropometric measurements were converted into height-for-age *Z* score (HAZ) and body mass index (BMI)-for-age *Z* score (BAZ) using WHO AnthroPlus (version 1.0.4). Children whose BAZ was below −2 standard deviation (SD) and whose BAZ was >−2 SD of the WHO standard were classified as wasted and normal, respectively. However, stunting was used as an indicator of chronic malnutrition and was defined as HAZ < −2 SD of the WHO standard [4, 34].

#### 2.5.2. Hemoglobin Measurement

The hemoglobin level was adjusted for the altitude of the town before defining anemia [5] by using the formula: Hob = −0.32 × (altitude in meters × 0.0033) + 0.22 × (altitude in meters × 0.0033) [16, 17]. Then, the Hob concentration of each participant was measured by pricking the tip of the finger with a sterile disposable lancet. The first and second drops of blood were wiped away, and the third drop of blood was allowed to enter the optical window of the microcuvette through capillary action and using a portable hemoglobinometer instrument (HemoCue 301+ analyzer, Sweden), which was recommended by the WHO for the use of field surveys [4, 10, 22, 35]. Two trained senior laboratory technologists and one senior laboratory technologist were involved when the discrepancy occurred between the two data collectors.

The cuvette holder was cleaned every day with alcohol or mild detergent (checking the operating manual) and the optical unit was cleaned, once a month with a HemoCue cleaner (cleaning swab), which comes together with the analyzer. Also, it is cleaned after 50 measurements or when an error message is shown. Hemoglobin readings were corrected for altitude by subtracting the expected normal increase in altitude proposed by the WHO based on the altitude of the town nearest to each school [33].

#### 2.5.3. Stool Examination

From each study participant, fresh stool samples were collected following the standard operating procedures (SOPs) in clean and labelled leak-proof stool cups. The stool specimens were transported in screw-capped cups in 10% formalin to the laboratory. Intestinal parasites were detected microscopically by direct saline wet mount. A saline wet mount was made by mixing a small quantity (about 2 mg) of feces in a drop of saline placed on a clean glass slide. Then, the smear stool was examined under a microscope. A saline wet mount was used for the detection of trophozoites and cysts in protozoa and eggs and larvae in helminths.

### 2.6. Study Variables

#### 2.6.1. Dependent Variables

The dependent variable includes anemia status (yes/no).

#### 2.6.2. Independent Variables

Sociodemographic characteristics of school children and parents or guardians are as follows: respondent age, sex, ethnicity, grade level of children, religion, marital status of parent, occupational status of parent, educational level of parent, and family size. Nutrition-related factors are as follows: stunting, wasted, underweight, taking tea and/or coffee within 30 minutes after a meal, and dietary diversification.

### 2.7. Measurements and Operational Definitions

  Anemia: hemoglobin level <11.5 g/dl for age ranges from 5 to 11 years (5, 9, 12)
  Mild Anemia: hemoglobin level of 11.0–11.4 g/dl for age ranges from 5 to 11 years (5, 9, 12)
  Moderate Anemia: hemoglobin level of 8.0–10.9 g/dl for age ranges from 5 to 11 years (5, 9, 12)
  Severe Anemia: hemoglobin level of <8.0 g/dl for age ranges from 5 to 11 years (5, 9, 12)
  Anemia: hemoglobin level below 12 g/dl for age ranges from 12 to 14 years (5, 9, 12)
  Mild Anemia: hemoglobin level of 11.0–11.9 g/dl for age ranges from 12 to 14 years (5, 9, 12)
  Moderate Anemia: hemoglobin level of 8.0–10.9 for age ranges from 12 to 14 years (5, 9, 12)
  Severe Anemia: hemoglobin level of <8.0 g/dl for age ranges from 12 to 14 years (5, 9, 12)
  Dietary Diversity Score: it was calculated from ten food groups, and children getting greater or equal to 7 food groups were classified as getting high dietary diversity; otherwise, they were considered as getting medium and low dietary diversity scores of 4–6 and less than or equal to 3, respectively [12]
  Intestinal Parasite Infection: the participant was recorded as positive for intestinal parasites if the stool sample examined by microscopy became positive for ova, cysts, or trophozoites of any parasites [36, 37]

### 2.8. Data Quality Assurance

A questionnaire was prepared in English and then translated into Afaan Oromo for appropriateness and easiness by linguistic language experts. The Afaan Oromo questionnaire was again translated back to the English language to verify the content validity of the original version. The training was given to data collectors and supervisors for two days on the method of anthropometric measurements, blood and stool sample collection and examination, ethical issues, and the purpose of the study. Hemoglobin measurement and stool microscopic examination were carried out following standard operating procedures (SOPs), and a HemoCue Hob 301 analyzer was used to determine the hemoglobin level. A direct stool examination using a saline smear was used to investigate the intestinal protozoa, eggs, and larvae of helminths. Daily monitoring was carried out during the data collection period. A pretest was conducted on 5% of students at primary schools in Iteya Town, which is located 25 kilometers away from the study area, and necessary modifications were made.

### 2.9. Data Processing and Analysis

The collected data were checked for completeness, coded, and entered into EpiData version 4.6 and then exported to SPSS for Windows version 26 for statistical analysis. Descriptive statistical analysis was used to compute the frequency and percentage of independent and dependent variables. Bivariable analyses were carried out by using a binary logistic regression model to select candidate variables for multivariable logistic regression analysis.

Variables with a *p* value <0.25 in the bivariable analysis were transferred to the multivariable analysis to control the effect of confounders. The strength of association was measured using adjusted odds ratios with a *p* value less than or equal to 0.05 together with 95% confidence intervals in the multivariable analysis and was considered significantly associated. Multicollinearity was also checked among selected variables by using a cut-off point of variance inflation factor <10. However, the result of VIF for all variables was found to be less than 10 (VIF = 5.62), which indicates there was no multicollinearity among variables. The Hosmer–Lemeshow model was used to check model fitness, and its *p* value was found to be 0.64. Finally, the results were presented in the form of tables, figures, and texts based on the obtained data.

## 3. Results

### 3.1. Sociodemographic Characteristics of the SAC

A total of 435 school-age children have participated in this study, making a response rate of 98.4%. Of the total, 227 (52.2%) were male. Participants ranged in age from 7 to 14 years, with a mean age of 10.77 and an SD of 2.21. Two hundred fifty-one (57.7%) SC were aged 7–11 years. Three hundred forty-two (78.6%) participants were Oromo in ethnicity. Two hundred thirty-one (53.1%) participants were in grades 1–4. The estimated mean family size of the participants was 5.20 and 1.662 SD, and 279 (64.1%) participants had 5 or more family members (Table 2).

### 3.2. Magnitude of Anemia among School-Age Children

**Figure 2:**
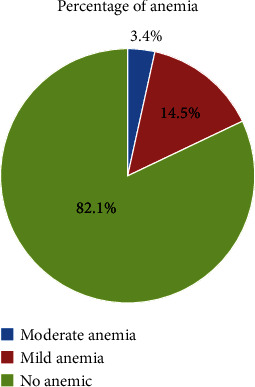
Magnitude of anemia among school-age children in public primary school students in Asella Town, Southeast Ethiopia, 2022.

The magnitude of anemia among SAC was 78 (17.9%) with a 95% CI (14.33, 21.47). The hemoglobin level of the SAC ranged from 10.1 g/dl to 15.4 g/dl with a mean (±SD) value of 12.78 ± 1.107 g/dl. Forty-eight (19.1%) SAC who had anemia were in the age group of 7–11 years, of whom 30 (16.3%) were in the age group of 12–14 years (Figure 2).

### 3.3. Anthropometric and Clinical Characteristics of School-Age Children

The stool samples were collected from 435 school-age children, of whom 32 (7.4%) were positive for intestinal parasites and the remaining 403 (92.6%) were negative. Hookworms (15 (46.9%)) and Ascaris lumbricoides (11 (34.4%)) were the predominant species. The mean height and weight of the SAC were 138 (±0.119) cm and 30.6 (±7.404) kg, respectively. The mean body mass index-for-age *Z* score (BAZ) was −0.88 (±1.321), and the mean height-for-age *Z* score (HAZ) was −0.72 (±0.888). Twenty-nine (6.7%) and 70 (16.1%) school-age children were stunted and underweight, respectively (Table 3).

### 3.4. Dietary Diversity Practice of School-Age Children

**Figure 3:**
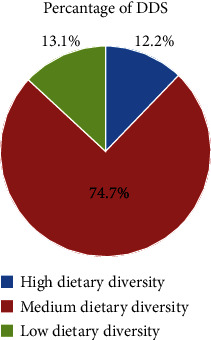
Dietary diversity score among school-age children in public primary school students in Asella Town, Southeast Ethiopia, 2022.

Regarding the dietary diversity score (DDS), the mean dietary diversity score of respondents was 5 (±1.373). Three hundred twenty-five (74.7%) had a medium dietary diversity score and consumed four up to six different food groups, followed by 53 (12.2%) SAC, who had a high dietary diversity score of seven and above food groups, and the rest of the respondent had a low DDS of three and below food groups. The minimum dietary diversity score of school-age children was 2 (consumed only two food groups), and the maximum dietary diversity score was 9 out of 10 food group items (Figure 3).

### 3.5. Factors Associated with Anemia among School-Age Children

In the bivariable analysis, father occupation, mother occupation, family size, mother educational status, father educational status, underweight, dietary diversification, and intestinal parasite were significantly associated with anemia among school-age children at *p* value less than 0.25. The result of the multivariable logistic regression model revealed that mother education, underweight, and intestinal parasites were found to be significantly associated with anemia among school-age children at a *p* value of ≤0.05 together with 95% confidence intervals.

Children who were positive for intestinal parasitic infections were 3.72 times more likely to be anemic than those who were negative for intestinal parasitic infections (AOR = 3.72, 95% CI: 1.50, 9.20). Students whose mothers had no formal education were 3.94 times more likely to be anemic than students whose mothers had secondary and above levels of education (AOR = 3.94, 95% CI: 1.89, 8.21). Underweight children were 3.83 times more likely to be anemic than normal weight children (AOR = 3.83; 95% CI: 1.98, 7.40) (Table 4).

## 4. Discussion

The overall magnitude of anemia among school-aged children identified in this study is 17.9%, with a 95% CI (14.33, 21.47). This study identified factors that established an association with anemia among school-aged children such as intestinal parasitic infections, maternal education, and being underweight.

The magnitude of the present study was in line with the study conducted in Gondar Town (15.5%) [12] and North-Western Morocco (16.2%) [38]. However, this result was lower than a study conducted in Indonesia (45.57%), Egypt (38.7%), Somaliland (23.1%), Arab Minch (37.3%), Pawe Town, Northwest Ethiopia (33.9%) [15, 16, 28, 39], and Gurage Zone (21.71%) [40]. The difference might be related to the difference in sample size used, study population, setting of the study area, time period gap among studies, geographical variation, and socioeconomic status. Currently, in Ethiopia, there are several interventions made by the Ministry of Health through health extension workers that might contribute their share to decreasing the burden of anemia among school children.

The magnitude of anemia in this study is higher than that in a study conducted in Northwest Ethiopia (7.6%) [41], Durbete Town, Northwest Ethiopia (10.7%) [29], and China (8.4%) [27]. The discrepancy may be attributed to the differences in socioeconomic, sociodemographic, and cultural behaviors, studied population, and study period and setting.

In this study, maternal education was found to be significantly associated with anemia in SC. Children whose mothers had no formal education were 3.94 times more likely to be anemic than children whose mothers had secondary and above levels of education. This finding is supported by the study conducted in Egypt, Somaliland, Gondar Town, and Pawe Town [12, 26, 28, 39]. This might be related to a low level of awareness among uneducated mothers to understand the nutritional requirements of children, easily follow the recommended child-feeding practices, and use diversified diets including iron and other micronutrients.

Underweight children were 3.83 times more likely to be anemic than normal weight children. Our finding was in agreement with studies conducted in Pawe Town, Benishangul-Gumuz Region [19]. This might be related to the fact that underweight is due to acute or chronic inadequate nutrition intake. In addition, not washing hands before eating may cause an acute bacterial or parasitic infection that would lead to diarrhea or malabsorption problems and acute undernutrition. Also, this might be due to the long-term effects of low iron intake and other micronutrient deficiencies.

School-age children infected with intestinal parasites are 3.72 times more likely to be anemic than noninfected children. This finding was supported by studies conducted in Somaliland, Filtu Town, Arba Minch, Gondar Town [13, 16, 19, 21], and Mihur Na Aklil District of Gurage Zone [40]. This may be because the majority of intestinal parasites, particularly hookworm and ascariasis, contribute to blood loss and/or red cell destruction and thus contribute to anemia. In addition, this might be due to nutritional competition, red blood cell destruction and feeding, and loss of appetite caused by worms. Additionally, these parasites directly invade and damage the intestinal mucosa wall, which impairs the absorption of nutrients.

### 4.1. Limitations of the Study

This study used a cross-sectional study design that does not show cause-and-effect relationships. Recall bias might have been introduced. There are a few limited reference materials used to compare similar studies in the same study area.

## 5. Conclusion

In this study, the magnitude of anemia was a mild public health problem among the SAC. Intestinal parasitic infections, maternal education, and being underweight were found to be the major factors that contributed to the development of anemia in the study area. Thus, health professionals should provide health education for mothers about child-feeding practices and consumption of dietary sources of iron to reduce the burden of anemia, and the Asella Town Health Office should strengthen nutrition education, good hygiene, and sanitation in the community and school.

The families of the study participants should focus on periodic deworming of their children, which is an essential intervention in children because intestinal parasites, particularly hookworm infection, lead to blood loss in the intestines, which, in turn, contributes to anemia. Asella Town Education Bureau should work on strengthening the schools to improve personal hygiene and environmental sanitation and control the transmission of these parasites, and school teachers should advise both children and their parents regarding the advantages of a balanced diet.

## Figures and Tables

**Table 1:** Double population proportions based on factors associated with anemia in school children in Asella Town, Oromia Regional State, Southeast Ethiopia, 2022.

Variables	Percent of outcome among unexposed (%)	Percent of outcome among exposed (%)	Sample size	Final sample size	Reference
Stunting	12.18	47.01	64	141	[32]
Underweight	16.33	41.35	116	256	[32]
Intestinal parasites	21.89	41.98	188	410	[32]
Maternal education	13.4	42.7	86	208	[26]

**Table 2:** Sociodemographic characteristics of SAC among public primary school students in Asella Town, Southeast Ethiopia, 2022.

Variables (*n* = 435)	Category	Number	Percent (%)
Mother education	No formal education	75	17.2
	Primary education	193	44.8
	Secondary and above	167	37.9

Father education	No formal education	44	10.1
	Primary education	131	30.1
	Secondary and above	260	59.8

Mother occupation	Housewife	263	60.5
	Daily laborer	71	16.3
	Merchant	33	7.6
	Government employee	68	15.6

Father occupation	Daily laborer	213	49
	Merchant	34	7.8
	Government employee	188	43.2

Religion	Muslim	198	45.5
	Orthodox	223	51.3
	Protestant	14	3.2

**Table 3:** Anthropometric and clinical characteristics of school-age children in public primary school students in Asella Town, Southeast Ethiopia, 2022.

Variables	Category	Number	Percent (%)
Intestinal parasite in stool	Yes	32	7.4
	No	403	92.6

Types of intestinal parasites (*n* = 32)	*Ascariasis lumbricoides*	11	34.4
	Hookworm	15	46.9
	Amebiasis	3	9.4
	Giardia	3	9.4

Underweight	Yes	70	16.1
	No	365	83.9

Stunting	Yes	29	6.7
	No	406	93.3

**Table 4:** Bivariable and multivariable analysis of selected variables associated with anemia among SAC in public primary school students in Asella Town, Southeast Ethiopia, 2022.

Variables (*n* = 435)	Anemic *n* (%)	No anemic *n* (%)	Crude OR (95% CI)	Adjusted OR (95% CI)	*p* value
*Father occupation*					
Daily laborer	43 (20.18)	170 (79.82)	1.55 (0.90, 2.65)	1.4 (0.73, 2.71)	0.98
Merchant	10 (22.72)	34 (77.28)	1.8 (0.79, 4.09)	2.5 (0.95, 6.52)	0.99
Government employee	25 (14)	153 (86)	1.00	1.00	

*Mother occupation*					
Housewife	31 (13.36)	201 (86.64)	0.74 (0.37, 1.47)	0.57 (0.26, 1.25)	0.52
Daily laborer	17 (26.98)	49 (73.02)	1.66 (0.75, 3.69)	1.03 (0.41, 2.58)	0.45
Merchant	16 (28.57)	40 (71.43)	1.91 (0.85, 4.33)	1.6 (0.62, 4.13)	0.79
Government employee	14 (17.28)	67 (82.72)	1.00	1.00	

*Intestinal parasite*					
Yes	13 (40.63)	19 (59.37)	**3.55 (1.67, 7.56)**	**3.72 (1.50, 9.20)** ^*∗*^	**0.04**
No	65 (16.13)	338 (83.87)	1.00	1.00	

*Dietary diversity*					
Low dietary diversity	11 (19.29)	46 (80.71)	2.29 (0.74, 7.12)	2.53 (0.70, 9.11)	0.97
Medium dietary diversity	62 (19.07)	263 (80.93)	2.26 (0.86, 5.92)	2.82 (0.93, 8.57)	0.91
High dietary diversity	5 (9.43)	48 (90.57)	1.00	1.00	

*Family size*					
≥5	44 (15.77)	235 (84.23)	0.67 (0.41, 1.11)	0.64 (0.36, 1.15)	0.83
<5	34 (21.79)	122 (78.21)	1.00	1.00	

*Underweight*					
Yes	27 (38.57)	43 (61.43)	**3.86 (2.19, 6.80)**	**3.83 (1.98, 7.40)** ^*∗*^	**0.01**
No	51 (13.97)	314 (86.03)	1.00	1.00	

*Mother education*					
No formal education	33 (44)	42 (56)	**4.40 (2.35, 8.21)**	**3.94 (1.89, 8.21)** ^*∗*^	**0.03**
Primary education	20 (10.25)	175 (89.76)	0.64 (0.34, 1.20)	0.53 (0.26, 1.06)	0.62
Secondary and above	25 (15.15)	140 (84.85)	1.00	1.00	

*Father education*					
No formal education	15 (44.11)	29 (55.89)	3.02 (1.48, 6.16)	1.3 (0.54, 3.17)	0.75
Primary education	25 (19.08)	106 (80.92)	1.38 (0.79, 2.40)	1.22 (0.63, 2.39)	0.87
Secondary and above	38 (14.62)	222 (85.38)	1.00	1.00	

^*∗*^Statistically significant at *p* ≤ 0.05; 1 = used as a reference category. The bold values indicate significant category of the variables at *p* value <0.05.

## Data Availability

All the datasets generated and/or analyzed during the study are included in the study and available from the corresponding author upon reasonable request.

## Abbreviations

AOR: Adjusted odds ratio
BSc: Bachelor of science
DDS: Dietary diversity score
BAZ: Body mass index (BMI)-for-age *Z* score
HAZ: Height-for-age *Z* score
Hob: Hemoglobin
IDA: Iron deficiency anemia
SAC: School-age children
SC: School children
SOPs: Standard operating procedures
SPSS: Statistical Package of Social Science
VIF: Variable inflation factors
WASH: Water hygiene and sanitation
WHO: World Health Organization.
